# Narrowing the *FOXF1* distant enhancer region on 16q24.1 critical for ACDMPV

**DOI:** 10.1186/s13148-016-0278-2

**Published:** 2016-11-03

**Authors:** Przemyslaw Szafranski, Carmen Herrera, Lori A. Proe, Brittany Coffman, Debra L. Kearney, Edwina Popek, Paweł Stankiewicz

**Affiliations:** 1 Department of Molecular and Human Genetics, Baylor College of Medicine, One Baylor Plaza, ABBR R809, Houston, TX 77030 USA; 2 Department of Pediatrics, University of New Mexico, Albuquerque, NM USA; 3 Department of Pathology, University of New Mexico, Albuquerque, NM USA; 4 Department of Pathology and Immunology, Baylor College of Medicine, Houston, TX USA

**Keywords:** Genomic imprinting, Transcriptional enhancer, Copy-number variants

## Abstract

**Background:**

Alveolar capillary dysplasia with misalignment of pulmonary veins (ACDMPV) is a rare lethal lung developmental disorder caused by heterozygous point mutations or genomic deletions involving *FOXF1* or its 60-kb tissue-specific enhancer region mapping 270 kb upstream of *FOXF1* and involving fetal lung-expressed long non-coding RNA genes and CpG-enriched sites. Recently, we have proposed that the *FOXF1* locus at 16q24.1 may be a subject of genomic imprinting.

**Findings:**

Using custom-designed aCGH and Sanger sequencing, we have identified a novel de novo 104 kb genomic deletion upstream of *FOXF1* in a patient with histopathologically verified full phenotype of ACDMPV. This deletion allowed us to further narrow the *FOXF1* enhancer region and identify its critical 15-kb core interval, essential for lung development. This interval harbors binding sites for lung-expressed transcription factors, including GATA3, ESR1, and YY1, and is flanked by the lncRNA genes and CpG islands. Bisulfite sequencing of one of these CpG islands on the non-deleted allele showed that it is predominantly methylated on the maternal chromosome 16.

**Conclusions:**

Substantial narrowing and bisulfite sequencing of the *FOXF1* enhancer region on 16q24.1 provided new insights into its regulatory function and genomic imprinting.

**Electronic supplementary material:**

The online version of this article (doi:10.1186/s13148-016-0278-2) contains supplementary material, which is available to authorized users.

## Introduction

Alveolar capillary dysplasia with misalignment of pulmonary veins (ACDMPV, MIM 265380) is a lethal neonatal developmental lung disorder characterized by reduced number of pulmonary capillaries, muscular thickening in small pulmonary arterioles, and abnormally situated pulmonary veins running alongside pulmonary arterioles [1, 2]. Patients with ACDMPV present with respiratory distress usually accompanied by pulmonary hypertension and often by extra-pulmonary anomalies [3, 4].

In the vast majority of patients with ACDMPV, heterozygous de novo point mutations or genomic deletion copy-number variants (CNVs) of *FOXF1* or its distant enhancer region on chromosome 16q24.1 have been identified [4–7]. FOXF1 is a transcription factor involved, among others, in maintaining the endothelial barrier through activation of the S1P/S1PR1 signaling required for the integrity of adherens junctions [8].

An ~60 kb distant tissue-specific enhancer region (chr16:86,212,040-86,271,919; hg19) located 270 kb upstream of *FOXF1* has been defined following alignment of ACDMPV causative deletion CNVs that did not include *FOXF1* [7]. This region encodes fetal lung-expressed long non-coding RNAs, *LINC01081* and *LINC01082*, and encompasses binding sites for numerous transcriptional regulators, including CEBP/p300, CTCF, and GLI2 [4, 7]. Both *LINC01081* and GLI2 have been shown to positively control *FOXF1* expression in human fetal lung fibroblasts [7, 9]. Recently, in vivo studies of the mouse syntenic region have shown that it likewise harbors a *Foxf1* enhancer [10]. Of note, 30 of 31 genomic deletions pathogenic for ACDMPV, for which parental origin was determined, occurred de novo on the maternal chromosome 16, suggesting genomic imprinting of the *FOXF1* locus [4]. Importantly, some of the GLI2-binding sites located in the enhancer region have been found to reside in a partially methylated CpG island, and their function has been shown to be methylation sensitive [7]. Moreover, the putative promoter region of the *LINC01081* gene also includes a CpG island that could potentially be involved in its epigenetic regulation.

Here, we present a patient (144.3) manifesting full phenotype of ACDMPV caused by a deletion CNV involving only a portion of the 60-kb enhancer region that enabled us to identify its critical 15-kb core interval.

### Patient

The patient was a full-term baby boy, appropriate for gestational age, born via normal spontaneous vaginal delivery with meconium-stained amniotic fluid to a 22-year-old, G2, P2 mother. Pregnancy was uncomplicated. The mother had prenatal care and denied use of tobacco, alcohol, or drugs. Neonatal resuscitation was routine. Birth weight was 3.6 kg. Apgar scores were 8 and 9 at 1 and 5 min of life, respectively.

The patient’s early neonatal course was unremarkable until approximately 8 h of life, when he developed respiratory distress associated with oxygen saturations in the 80’s on room air, prompting admission to the neonatal intensive care unit (NICU). Chest X-ray revealed small bilateral pneumothoraxes and was not consistent with meconium aspiration syndrome. Echocardiogram showed patent foramen ovale with moderate right to left shunting, small patent ductus arteriosus with little flow into the pulmonary artery, mildly dilated right ventricle, mildly dilated right atrium, and severe pulmonary hypertension (3/4 systemic) while on inhaled nitric oxide (iNO) via nasal cannula at 12 h of life.

Upon admission to the NICU, the patient was started on iNO, initially via high flow nasal cannula without significant improvement prompting intubation and mechanical ventilation, first conventional followed by high-frequency oscillatory ventilation (HFOV) at approximately 24 h of life. The patient was transferred to HFOV plus iNO because of worsening hypoxemia complicated by systemic hypotension. Follow-up CXR showed well-expanded lungs and resolution of bilateral pneumothoraxes. The patient did not show significant improvement despite good alveolar recruitment on HFOV, optimization of systemic blood pressure with vasopressor medication, and iNO therapy. At approximately 48 h of life, he was placed on VV extracorporeal membrane oxygenation (ECMO) due to refractory hypoxemic respiratory failure. He remained relatively stable for 4 days on ECMO until he developed a right pneumothorax, which required chest tube placement. On day 7 of the ECMO run, the patient developed a left pneumothorax complicated by large hemothorax, pulmonary hemorrhage, disseminated intravascular coagulation, and tamponade physiology, including severe metabolic/lactic acidosis despite maximal ECMO and vasopressor support. On day 8 of the ECMO run, day 10 of life, the family decided to withdraw support given the patient’s severe clinical deterioration and grim prognosis.

Histopathological evaluation at autopsy confirmed the diagnosis of ACDMPV. The major pathological findings in the lungs included congested pulmonary veins adjacent to pulmonary arteries, thickened alveolar septae with decreased numbers of centrally located capillaries, and arteriolar hypertrophy of lungs.

## Material and methods

### DNA isolation and sequencing

Blood and FFPE lung samples were obtained after informed consent. DNA had been extracted from peripheral blood using Gentra Purgene Kit (Qiagen, Germantown, MD) and from lung tissue using DNaesy Blood & Tissue Kit (Qiagen). PCR products were treated with ExoSAP-IT (USB, Cleveland, OH) and directly sequenced by the Sanger method. Reference sequences were downloaded from the UCSC Genome Browser (NCBI build 37/hg19, http://genome.ucsc.edu). Sequences were assembled using Sequencher v4.8 (Gene Codes, Ann Arbor, MI).

### Array CGH and deletion analysis

Array CGH was done using custom-design 16q24.1 region-specific 4 × 180 K microarrays (Agilent, Santa Clara, CA) according to manufacturer protocol. Deletion junctions were amplified using LA Taq polymerase (Takara Bio., Madison, WI). Parental origin of the deletion-bearing chromosome was determined following identification of informative SNPs and microsatellite polymorphism in a region of parental chromosome 16 corresponding to patient’s deletion.

### Bisulfite sequencing

Bisulfite modification of DNA was performed using the EZ DNA Methylation-Lightning Kit (Zymo Research, Irvine, CA). Human Methylated and Non-methylated DNA Set (Zymo Research) was used to assess the efficiency of bisulfite-mediated conversion of DNA. Primers for PCR amplification of bisulfite-treated DNA (updelF: 5′-TTAGTTGGGGTTTATAAATTAGGTATTG-3′ and updelR1: 5′-AAACATTTCAAATAAATCTTTTAATTCC-3′) were designed using a MethPrimer software (http://urogene.org/methprimer/index1.html). PCR was performed in a 25-μL reaction mixture containing 200 ng of bisulfite-treated DNA, and 0.75 units of Taq polymerase (Invitrogen, Carlsbad, CA), applying 30 cycles of the three-step incubation for 30 s at 94 °C, 30 s at 55 °C, and 1 min at 72 °C. The PCR product was purified from unincorporated primers and nucleotides using the MinElute PCR Purification Kit (Qiagen), and T-vector cloned in DH5α cells using the pGEM-T Easy Vector System (Promega, Madison, WI). Plasmid minipreps were prepared from 12 randomly selected transformant colonies using the QIAprep Spin Miniprep Kit (Qiagen) and Sanger sequenced using SP6 universal primer.

## Results

**Fig. 1:**
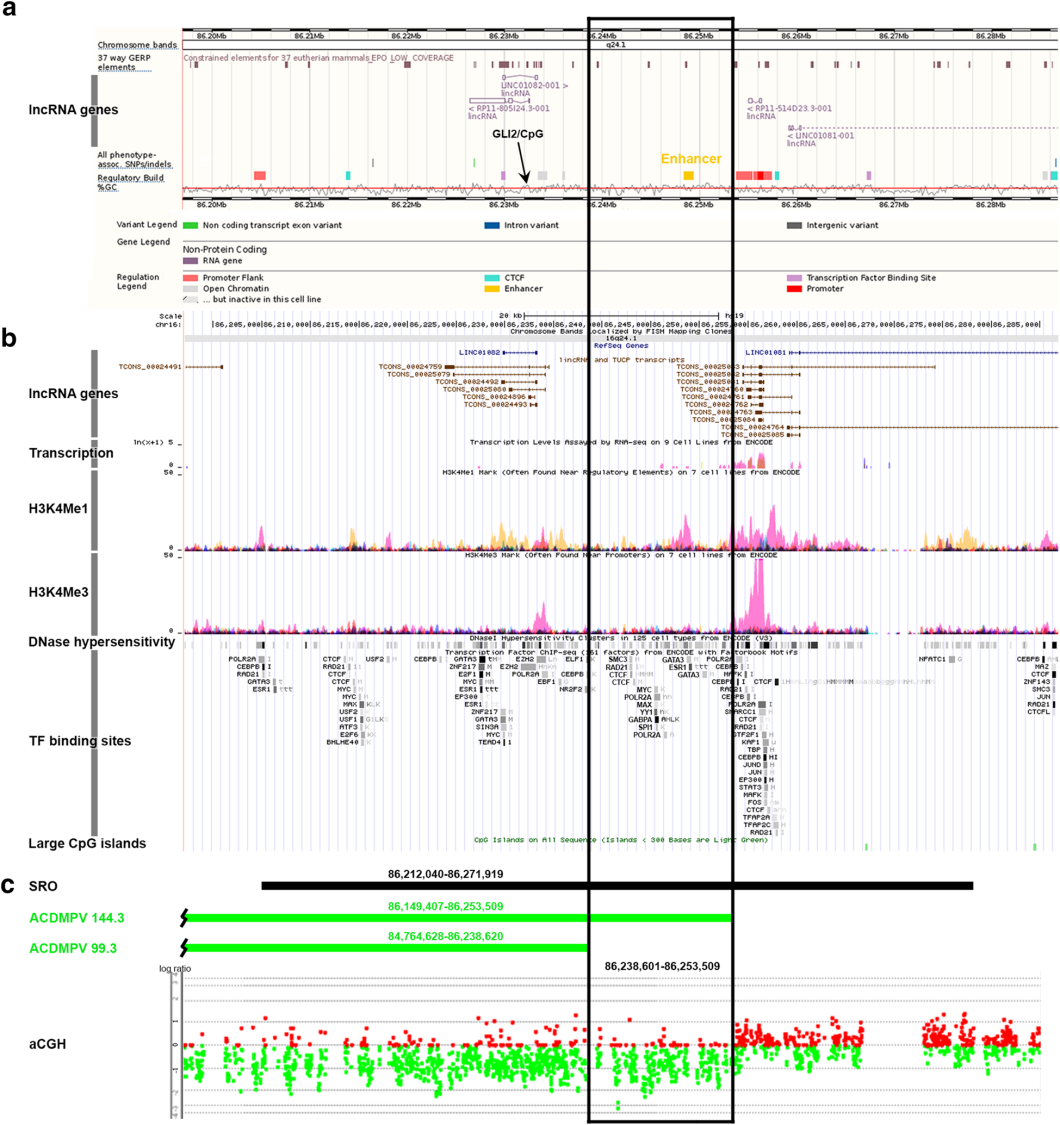
Schematic representation of a protein-coding gene desert-region on 16q24.1 containing the *FOXF1* upstream enhancer. **a** ENCODE and **b** RefSeq annotations of the region (GRCh37/hg19). **c** Delineation of the enhancer 15-kb critical interval (*black frame*) by array CGH. SRO ~60 kb smallest deletion overlap (see Additional file 1: Figure S3)

Sanger sequencing of the coding portion of *FOXF1* did not reveal any single nucleotide or indel variant. Array CGH identified a 104-kb heterozygous deletion CNV upstream of *FOXF1*, partially overlapping the 60-kb enhancer region (Fig. 1). Sequencing of the PCR-amplified deletion junction showed that the deletion proximal breakpoint maps at chr16:86,149,407 within a simple repeat (CATATA)_n_ and the distal breakpoint maps at chr16:86,253,509 within a unique sequence (Additional file 1: Figure S1). There was no microhomology at the breakpoints. Parental studies revealed that the deletion arose on the maternal chromosome 16 (Additional file 1: Table S1, Figure S2). We did not detect by PCR any evidence of low-level somatic mosaicism in the parental DNA samples from the peripheral blood, indicating that the deletion arose de novo.

Comparison of the region deleted in the described patient 144.3 with that deleted in patient 99.3 who was diagnosed with a late onset ACDMPV (chr16:84,764,628-86,238,601) [10] enabled us to define the 15-kb critical enhancer interval chr16:86,238,601-86,253,509. This interval harbors sequences predicted by the encyclopedia of DNA Elements (ENCODE) project to function as transcriptional enhancer (Fig. 1a). It is characterized in the human lung fibroblasts by increased DNase hypersensitivity, significantly increased ratio of histone H3 Lys 4 monomethylation (H3K4Me1, a typical marker of active transcriptional enhancers) compared with trimethylation (H3K4Ma3, typical for promoters), and the presence of histone acetylation H3K27Ac (typical for active enhancers) (Fig. 1b). In addition to harboring the CTCF binding sites, the identified 15-kb region contains also the clustered binding sites for multiple transcription factors expressed in the lung (BioGPS, http://biogps.org), including GATA3, ESR1, GABPA, YY1, SMC3, RAD21, MYC, MAX, and SPI1 that might explain its function as a positive regulator of transcription (Fig. 1b).

The 15-kb interval does not encompass clearly defined CpG islands. However, it is possible that it harbors the CpG island-free differential methylation region (DMR) for the locus. Nonetheless, this region neighbors a partially methylated CpG island overlapping the GLI2-binding sites that have been shown previously to regulate in vitro expression of *FOXF1* [7]. We have bisulfite sequenced this CpG island in the lung DNA from patients 144.3 and 60.4 with the *FOXF1* upstream enhancer region deleted on the maternal chromosome 16 [7] from two normal lung samples (Additional file 1: Figure S3). Interestingly, we have found that although the methylation of this CpG island is variable in the patient and normal lungs, the extent of methylation depends on the parental origin of chromosome 16 with 4 of 11 CpG sites analyzed being preferentially methylated on the maternal allele overlapping the GLI2-binding sites.

## Discussion

Using customized region-specific high-resolution array CGH, we have identified a functionally essential 15-kb core interval in the *FOXF1* upstream enhancer region. In fetal lung fibroblasts, this interval features histone modifications typically found in active enhancers and contains sites for binding of numerous transcription factors. In addition to binding CTCF, which can mediate long-range chromatin interactions, of note are the binding sites for GATA3, ESR1, and YY1. GATA3 is a pioneer transcription factor recruiting SWI/SNF chromatin remodeling complex. It is essential for chromatin modification that makes it accessible for ESR1 and other transcription factors [11]. Interestingly, ESR1 has been found to be involved in fetal lung development [12] and mediation of long-range chromatin looping that can position enhancer-bound transcription factors in the proximity to their target promoter [13]. YY1 is a multifunctional transcription factor of the Polycomb family also required during lung morphogenesis. It is expressed in lung mesenchyme [14] as well as in epithelium where it targets SHH expression known to regulate *FOXF1* expression.

The deletion in patient 144.3 occurred on maternal chromosome 16, similarly to 30 of 31 other deletions pathogenic for ACDMPV in this region [4] (Additional file 1: Figure S3), further indicating that the *FOXF1* locus on 16q24.1 is imprinted. However, the 15-kb core interval does not contain any well-defined CpG island, and other chromatin modifications such as histone methylation or acetylation can contribute to genomic imprinting of this genomic region.

We have elected to bisulfite sequence the CpG island overlapping the GLI2-binding sites located proximally to the 15-kb interval that has been shown to be partially methylated [7]. We found that the extent of methylation of this CpG island was variable but overall higher on maternal chromosome 16, with four out of 11 CpG dinucleotides being preferentially maternally methylated. Interestingly, a small maternal DMR involving the *Mcts2* retrogene mapping in an intron of the multi-exonic host *HM13* gene in mice has been shown to regulate *HM13* expression in a parent-of-origin manner, demonstrating that discrete regions can bring about imprinting [15]. This finding supports a model of partial maternal imprinting of the *FOXF1* locus where loss of its paternal copy is embryonic lethal and loss of the maternal copy results in ACDMPV [4]. However, most recently, no evidence of differential methylation was found in the *FOXF1* locus using Illumina Infinium HumanMethylation450 BeadChip Array analysis [16], indicating that other epigenetic mechanism can be responsible for genomic imprinting of this locus.

The reported deletion does not include the lncRNA *LINC01081* gene, which we have shown to positively regulate *FOXF1* expression in vitro [10], suggesting that *LINC01081* may be less critically involved in *FOXF1* regulation in developing lungs than previously thought. Alternatively, the described 15-kb region may regulate the expression of *LINC01081* or some of its shorter isoforms (TCONS_00024760-3, TCONS_00025081, 2, 4, 5), or eRNAs.

We conclude that the described 15-kb sequence located between *LINC01082* and *LINC01081* encompasses the enhancer interval essential for regulation of the expression of *FOXF1* in the fetal lungs that functions likely as a scaffold for transcription factors and mediator of chromatin looping juxtaposing these factors, the *FOXF1* promotor, and/or the lncRNA genes. The putative imprinting center for the *FOXF1* locus on chromosome 16q24.1 may be located either more centromeric (e.g., in the nearby CpG island with GLI2-binding sites), telomeric (e.g., in the CpG island overlapping with putative promoter of *LINC01081*), or within this interval but not involving the CpG island. Alternatively, mechanism(s) other than CpG methylation can be responsible for the observed genomic imprinting. High-resolution approaches, e.g., targeted DNA methylation analysis by next generation sequencing (methyl-Seq), can help to unravel the molecular bases of this phenomenon.

## Additional file

Additional file 1: Figure S1. Chromatopherogram of the sequence across the 144.3 deletion junction. Note that GAA nucleotides inserted at the junction of the deleted fragment (chr16:86,238,601-86,253,508). Table S1. Determination of the maternal origin of the deletion upstream of *FOXF1* identified in the described ACDMPV patient 144.3. Figure S2. Chromatopherograms of DNA sequences containing the informative SNPs and microsatellite used to determine the parental origin of the deletion in patient 144.3. Figure S3. Methylation status of the CpG island mapping proximally to the 15 kb critical interval of the *FOXF1* enhancer region. Filled circles (chr16:86,232,414-86,232,582) represent methylated CpGs. The inset shows average methylation status of each CpG. CpGs 1, 2, 6 and 11 are more often methylated on maternal chromosome 16. Figure S6. Schematic representation of chromosome 16q24.1 deletions pathogenic for ACDMPV. Thirty two of 33 deletion CNVs, which occurred on maternal chromosome 16, are shown in red, the deletion on paternal chromosome is shown in blue, and deletions, for which parental origin could not be determined, are shown in black. Numbers refer to ACDMPV cases. Locations of deletion breakpoints (BPs) are indicated by names of flanking repetitive elements. SRO, smallest deletion overlap delineating upstream enhancer region, unk unknown sequence, uniq unique sequence. Previously published deletions are from the reference [4]. (DOCX 2158 kb)

## Abbreviations

ACDMPV: Alveolar capillary dysplasia with misalignment of pulmonary veins
CNV: Copy-number variant
ECMO: Extracorporeal membrane oxygenation
ENCODE: Encyclopedia of DNA Elements
HFOV: High-frequency oscillatory ventilation
iNO: Inhaled nitric oxide
NICU: Neonatal intensive care unit

## References

[1] Langston C (1991). Misalignment of pulmonary veins and alveolar capillary dysplasia. Pediatr Pathol.

[2] Sen P, Thakur N, Stockton DW, Langston C, Bejjani BA (2004). Expanding the phenotype of alveolar capillary dysplasia (ACD). J Pediatr.

[3] Bishop NB, Stankiewicz P, Steinhorn RH (2011). Alveolar capillary dysplasia. Am J Respir Crit Care Med.

[4] Szafranski P, Gambin T, Dharmadhikari AV, Akdemir KC, Jhangiani SN, Schuette J (2016). Pathogenetics of alveolar capillary dysplasia with misalignment of pulmonary veins. Hum Genet.

[5] Stankiewicz P, Sen P, Bhatt SS, Storer M, Xia Z, Bejjani BA (2009). Genomicand genic deletions of the FOX gene cluster on 16q24.1 and inactivating mutations of *FOXF1* cause alveolar capillary dysplasia and other malformations. Am J Hum Genet.

[6] Sen P, Yang Y, Navarro C, Silva I, Szafranski P, Kolodziejska KE (2013). Novel *FOXF1* mutations in sporadic and familial cases of alveolar capillary dysplasia with misaligned pulmonary veins imply a role for its DNA binding domain. Hum Mutat.

[7] Szafranski P, Dharmadhikari AV, Brosens E, Gurha P, Kolodziejska KE, Zhishuo O (2013). Small noncoding differentially methylated copy-number variants, including lncRNA genes, cause a lethal lung developmental disorder. Genome Res.

[8] Cai Y, Bolte C, Le T, Goda C, Xu Y, Kalin TV, Kalinichenko VV. FOXF1 maintains endothelial barrier function and prevents edema after lung injury. Sci Signal. 2016;9:ra40. 10.1126/scisignal.aad1899 27095594

[9] Szafranski P, Dharmadhikari AV, Wambach JA, Towe CT, White FV, Grady RM (2014). Two deletions overlapping a distant *FOXF1* enhancer unravel the role of lncRNA LINC01081 in etiology of alveolar capillary dysplasia with misalignment of pulmonary veins. Am J Med Genet A.

[10] Seo H, Kim J, Park G-H, Kim Y, Cho SW (2016). Long-range enhancers modulate *Foxf1* transcription in blood vessels of pulmonary vascular network. Histochem Cell Biol.

[11] Takaku M, Grimm SA, Shimbo T, Perera L, Menafra R, Stunnenberg HG (2016). GATA3-dependent cellular reprogramming requires activation-domain dependent recruitment of a chromatin remodeler. Genome Biol.

[12] Gortner L, Shen J, Tutdibi E (2013). Sexual dimorphism of neonatal lung development. Klin Padiatr.

[13] Theodorou V, Stark R, Menon S, Carroll JS (2013). GATA3 acts upstream of FOXA1 in mediating ESR1 binding by shaping enhancer accessibility. Genome Res.

[14] Bérubé-Simard FA, Prudhomme C, Jeannotte L (2014). YY1 acts as a transcriptional activator of *Hoxa5* gene expression in mouse organogenesis. PLoS ONE.

[15] Wood AJ, Schulz R, Woodfine K, Koltowska K, Beechey CV, Peters J, Bourc’his D, Oakey RJ (2008). Regulation of alternative polyadenylation by genomic imprinting. Genes Dev.

[16] Joshi RS, Garg P, Zaitlen N, Lappalainen T, Watson CT, Azam N (2016). DNA methylation profiling of uniparental disomy subjects provides a map of parental epigenetic bias in the human genome. Am J Hum Genet.

