# Supplementary material for: Narrowing the FOXF1 distant enhancer region on 16q24.1 critical for ACDMPV
Source: Clin Epigenetics. 2016 Nov 3;8:112. doi: 10.1186/s13148-016-0278-2 (PMC5093964; doi:10.1186/s13148-016-0278-2)
Supplement: Additional file 1: Figure S1. — Chromatopherogram of the sequence across the 144.3 deletion junction. Note that GAA nucleotides inserted at the junction of the deleted fragment (chr16:86,238,601-86,253,508). Table S1. Determination of the maternal origin of the deletion upstream of FOXF1 identified in the described ACDMPV patient 144.3. Figure S2. Chromatopherograms of DNA sequences containing the informative SNPs and microsatellite used to determine the parental origin of the deletion in patient 144.3. Figure S3. Methylation status of the CpG island mapping proximally to the 15 kb critical interval of the FOXF1 enhancer region. Filled circles (chr16:86,232,414-86,232,582) represent methylated CpGs. The inset shows average methylation status of each CpG. CpGs 1, 2, 6 and 11 are more often methylated on maternal chromosome 16. Figure S6. Schematic representation of chromosome 16q24.1 deletions pathogenic for ACDMPV. Thirty two of 33 deletion CNVs, which occurred on maternal chromosome 16, are shown in red, the deletion on paternal chromosome is shown in blue, and deletions, for which parental origin could not be determined, are shown in black. Numbers refer to ACDMPV cases. Locations of deletion breakpoints (BPs) are indicated by names of flanking repetitive elements. SRO, smallest deletion overlap delineating upstream enhancer region, unk unknown sequence, uniq unique sequence. Previously published deletions are from the reference [4]. (DOCX 2158 kb) [file 13148_2016_278_MOESM1_ESM.docx]

**Additional files**

**Narrowing the *FOXF1* distant enhancer region on 16q24.1 implicated in ACDMPV**

Przemyslaw Szafranski^1^, Carmen Herrera^2^, Lori A. Proe^3^, Brittany Coffman^3^,

Debra L. Kearney^4^, Edwina Popek^4^, Paweł Stankiewicz^1,*^

^1^Department of Molecular and Human Genetics, Baylor College of Medicine, Houston, Texas, USA; ^2^Department of Pediatrics, University of New Mexico, Albuquerque, New Mexico, USA; ^3^Department of Pathology, University of New Mexico, Albuquerque, New Mexico, USA; ^4^Department of Pathology and Immunology, Baylor College of Medicine, Houston, Texas, USA

**Additional file 1**

**
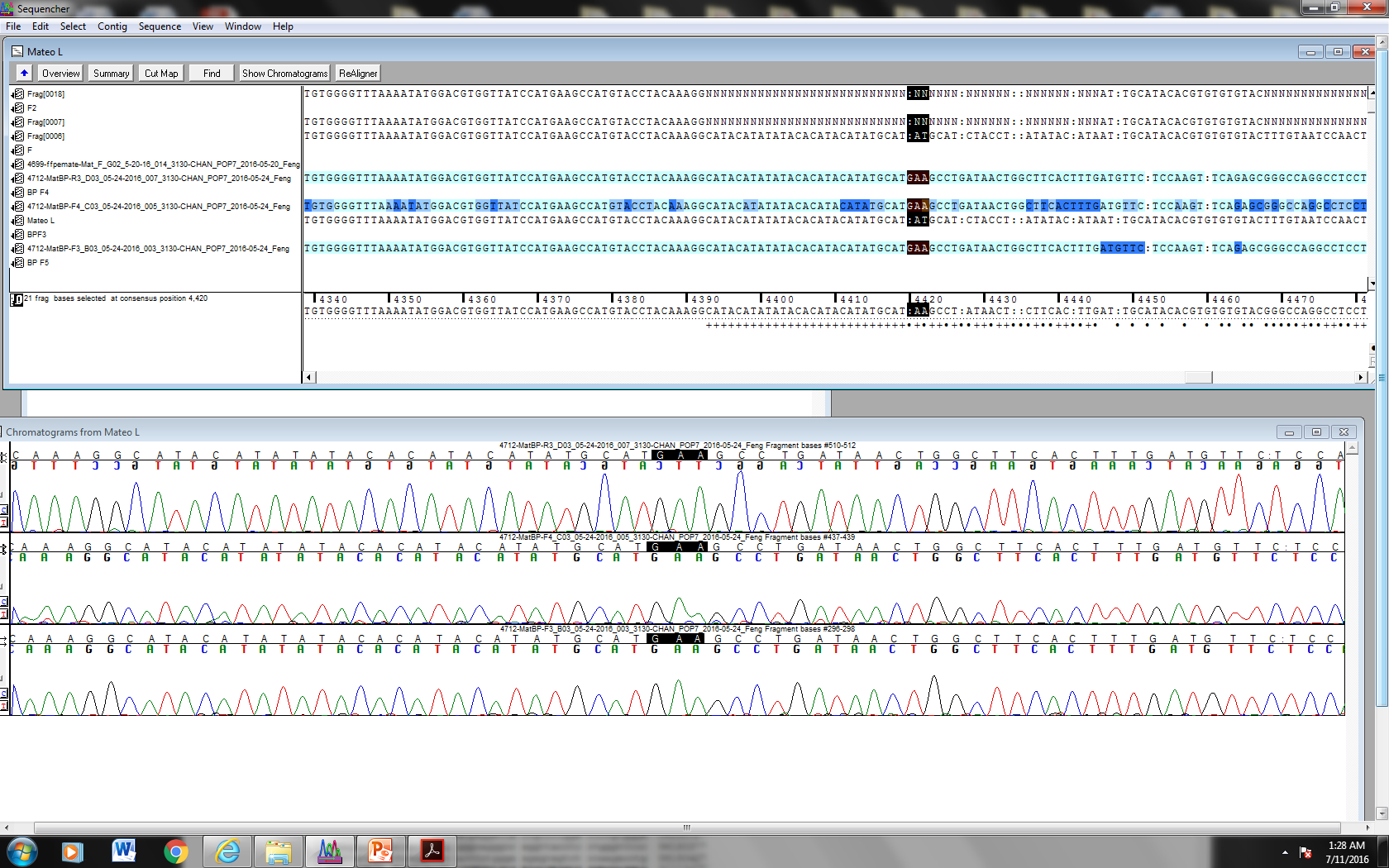
**

**Figure S1.** Chromatopherogram of the sequence across the 144.3 deletion junction. Note that GAA nucleotides inserted at the junction of the deleted fragment (chr16:86,238,601-86,253,508).

**Additional file 2**

**Table S1.** Determination of the maternal origin of the deletion upstream of *FOXF1* identified in the described ACDMPV patient 144.3.

|  | **Father 144.1** | **Mother 144.2** | **Proband 144.3** |
| --- | --- | --- | --- |
| Microsatellite: 26xAC  chr16:86,165,989-86,166,041 | (CA)_26_/(CA)_12_ | (CA)_26_/(CA)_26_ | (CA)_12_ |
| rs12102482: T/C  chr16:86,231,288 | C/T | T/T | C |
| rs11117465: C/T  chr16:86,231,329 | T/C | C/C | T |

**Figure S2.** Chromatopherograms of DNA sequences containing the informative SNPs and microsatellite used to determine the parental origin of the deletion in patient 144.3.

**Additional file 3**

**Figure S3.** Filled circles (chr16:86,232,414-86,232,582) represent methylated CpGs. The inset shows average methylation status of each CpG. CpGs 1, 2, 6 and 11 are more often methylated on maternal chromosome 16.

**Additional file 4**

**Figure S4.** Schematic representation of chromosome 16q24.1 deletions pathogenic for ACDMPV. Thirty two of 33 deletion CNVs, which occurred on maternal chromosome 16, are shown in *red*, the deletion on paternal chromosome is shown in *blue*, and deletions, for which parental origin could not be determined, are shown in *black*. Numbers refer to ACDMPV cases. Locations of deletion breakpoints (BPs) are indicated by names of flanking repetitive elements. SRO, smallest deletion overlap delineating upstream enhancer region, *unk* unknown sequence, *uniq* unique sequence. Previously published deletions are from the reference [4].
